# Two Cases of Diseased Antrum

**Published:** 1899-03

**Authors:** W. H. M’Cutcheon

**Affiliations:** Brooklyn, N. Y.


### TWO CASES OF DISEASED ANTRUM.[1]

BY W. H. M'CUTCHEON, D.D.S., BROOKLYN, N. Y.

I TAKE pleasure in reporting this evening two cases of antral disease. The first was a Miss H., aged nineteen, who came in April, 1895. Her difficulty was an ulcerated right superior second bicuspid, which I had treated and filled in October, 1894. The root being filled with gutta-percha, I opened and treated with a five-per-cent. solution of chloride of zinc, and sealed the tooth with

[1] Read before The New York Institute of Stomatology, November 1, 1898.

gutta-percha, dismissing the patient for a week. At the end of that time she came and complained of severe neuralgia, extending to the region of the temple. I opened the tooth, which resulted in relief, though there was no pus present. I treated with a three-per-cent. solution of hydrogen dioxide, leaving loose cotton in the cavity, and dismissed the patient for one day, at the end of which time I extracted the tooth under protest, the patient complaining of severe neuralgia. I asked her to call again in two days, that I might syringe the socket, which I did with phenol sodique in solution of a drachm to the ounce. She reported relief from pain. After an interval of two weeks the patient returned, with severe neuralgia in same vicinity, and with first bicuspid sore to pressure. I opened the tooth, and found a partially devitalized pulp. I applied arsenic fibre and dismissed her till the next day, when I removed the pulp intact, filled canals, and asked the patient to call again in two weeks. She reported before two weeks, complaining of renewed neuralgia, and requested removal of first bicuspid, which I refused. The patient called on two other dentists, and both refused to extract the tooth. One of the doctors said that the entire trouble came from the right superior lateral incisor, which was, as far as I could see, not affected at all. The patient again resumed treatment under my charge, and, after exhausting all methods to alleviate the pain, I was about to give up the case, when I met Dr. Brockway and stated the circumstances. He called at my office April 12, 1896, and after examination we agreed that the first bicuspid had curvature of the root at the apical region, and decided to amputate the same. Dr. Brockway applied chloride of ethyl in spray, and with a large rose bur I drilled in the vicinity of the apex of the palatal root, through the alveolus, syringing with tepid carbolic solution of water, and inserted a tent of cotton with dressing of oil of eucalyptus and iodoform.

The next day, at 2.30 A.M., the patient reported at my office, and said the tooth must come out. I extracted it under protest. The patient again reported in about a month, complaining of throbbing pains in the malar region. After examination with electric mouth-lamp, I diagnosed the case as antrum disease. Opening the right superior first molar, I attempted to reach the antrum through the anterior buccal root, but finding that I could not, I extracted the tooth, and drilled up through the socket into the floor of the antrum, through which pus exuded immediately. I syringed with a three-per-cent. solution of hydrogen dioxide, which effervesced through right nostril and also by way of posterior nares, giving immediate relief as far as throbbing was concerned. I had Miss H. call twice daily, and used either hydrogen dioxide or phenol sodique in solution as an injection. In the summer of 1896 I inserted a silver drainage-tube, and asked her to spray the cavity herself with phenol sodique in solution. At the end of the summer she reported as being perfectly free from pain, and she has continued so up to the present time.

The second case, Mrs. W., aged fifty-eight, presented in April, 1898. Her difficulty was an ulcerated left superior second bicuspid, which contained a large contour gold filling. I drilled through this filling, and found a putrescent pulp, which I removed, and syringed out the canal with hydrogen dioxide, which partially exuded through the fistula, though the major portion went into the antrum. I attempted to treat the antrum through the root of the second bicuspid, but finding the diameter of the canal so small as to retard the exit of the solution, I extracted the tooth and sprayed through the socket twice daily. In the mean time the sixth-year molar had started to elongate, and upon opening it I found the contents of the palatine and the posterior buccal roots alive. I devitalized these and filled the three canals with gutta-percha. The patient soon complained of severe neuralgia in the vicinity of the posterior portion of this tooth, so I again opened the tooth and removed the gutta-percha from the posterior buccal and palatine canals, but there was no alleviation of pain.

I was called upon to extract the sixth-year molar, and did so, finding the posterior buccal root penetrating the floor of the antrum. I now had the two openings into the antrum, one from the socket of the second bicuspid root, and one from the socket of the posterior buccal root of the molar, through both of which I syringed, twice daily, phenol sodique, leaving tents of cotton in both sockets containing solution of oil of eucalyptus and iodoform.

I continued this treatment for one month till all odor and discharge of pus had ceased, then I gave patient carbolic solution (five minims to the ounce) to use as a spray herself with a syringe. I asked her to introduce the tents of cotton containing sweet oil into the posterior orifice, having in the mean time allowed the bicuspid socket to close. The third day after she called and complained of frightful sneezing spells, and gave me a large pledget of cotton which had been expelled from the left nostril, and had evidently been one of the tents. As this happened several times I prohibited her from using cotton as a tent, and vulcanized a little affair resembling an artist's flat-headed tack, which I directed her to insert during meal-times only. She reported in September, when I lacerated the walls of the socket to hasten granulation, the antrum being healed, and she has had no further trouble. I prefer something of the character of the little vulcanized plug referred to to either cotton tents or a drainage-tube in the treatment of these cases.

